# Evaluating the Integration of Genomics into Cancer Screening Programmes: Challenges and Opportunities

**DOI:** 10.1007/s40142-019-00162-x

**Published:** 2019-05-18

**Authors:** Sarah Briggs, Ingrid Slade

**Affiliations:** 1 Wellcome Centre for Human Genetics, Nuffield Department of Medicine, University of Oxford, Roosevelt Drive, Oxford, OX3 7BN UK; 2 Wellcome Centre for Ethics and Humanities and Ethox Centre, Nuffield Department of Population Health, Big Data Institute, Li Ka Shing Centre for Health Information and Discovery, University of Oxford, Old Road Campus, Oxford, OX3 7LF UK

**Keywords:** Cancer, Screening, Cancer genomics, Population health, Genetic susceptibility, Public health

## Abstract

**Purpose of Review:**

As the costs of genomic testing have fallen, and our understanding of genetic susceptibility to cancers has grown, there has been increasing interest in incorporating testing for cancer susceptibility genes, and polygenic risk estimates, into population cancer screening. A growing body of evidence suggests that this would be both clinically and cost-effective. In this article, we aim to explore the frameworks used to evaluate screening programmes, evaluate whether population screening for cancer susceptibility can be assessed using these standards, and consider additional issues and outcomes of importance in this context.

**Recent Findings:**

There are tensions between traditional approaches of genetic testing (utilising tests with high sensitivity and specificity) and the principles of population screening (in which the screening test typically has low specificity), as well as the frameworks used to evaluate the two. Despite the existence of many screening guidelines, including consensus papers, these often do not align fully with broader considerations of genetic test evaluation. Population screening for genetic risk in cancer shifts the focus from diagnostics to prognostication and has wider implications for personal and familial health than existing screening programmes. In addition, understanding of the prevalence and penetrance of cancer susceptibility genes, required by many screening guidelines, may only be obtainable through population-level testing; prospective multi-disciplinary research alongside implementation will be essential.

**Summary:**

Appropriate evaluation of genetic screening for cancer risk will require modification of existing screening frameworks to incorporate additional complexity of outcomes and population values. As evidence supporting population screening for cancer susceptibility mounts, development of an appropriate evaluative framework, and expansion of public dialogue will be key to informing policy.

## Introduction

As our understanding of genetic susceptibility to complex diseases, such as cancer, has grown over the last two decades, so too have calls from the scientific and clinical community for a broader approach to genetic testing, utilising this knowledge for “population” level benefit [1••]. The number of identified cancer susceptibility genes (CSGs) for high and moderate penetrance cancer syndromes is gradually expanding, and in addition, the identification of many common risk loci for cancer, discovered through genome-wide association studies (GWAS), has facilitated the development of clinically useful polygenic risk scores (PRS) [2–4]. These summarise risk conferred by many common variants, and facilitate stratification of the general population into levels of disease risk, enabling preventive measures such as screening to be targeted to higher risk groups. PRS do not currently have the power to define individual risk, but rather allocate individuals to a tier of risk within the population [5••]. There is a growing interest in incorporating both high and moderate penetrance CSGs and PRS into routine clinical care, in screening, diagnosis, prognostication and prevention.

Clinically, testing for CSGs has been limited to populations with high prior risk, i.e. with a strong family history, or cancer patients with suggestive risk factors for hereditary cancers. A growing body of evidence and opinion supports the introduction of *population screening* for CSGs through the application of screening tests in organised screening programmes [1••]. Population-based screening for CSGs has been trialled in select populations already, mainly BRCA screening in the Ashkenazi-Jewish community amongst whom there is a high prevalence of founder mutations [6–8]. In the UK, population screening for *BRCA1/BRCA2* mutations in the Ashkenazi-Jewish population detected 56% more cases than family-history-based testing [7], had high acceptability [9] and is highly cost-effective even when Ashkenazi-Jewish ancestry is limited to one grandparent [10]. Alongside this, there is increasing evidence supporting stratification of existing cancer screening programmes by risk (including genetic risk determined by both CSGs and PRS), to optimise patient outcomes, reduce over-diagnosis and better utilise limited resources [2, 3, 11–14].

However, genetic screening for cancer risk on a population level shifts the paradigms of both genetic testing and screening used in healthcare to date [15, 16]. The focus traditionally of genetic testing has been on the diagnosis of high-risk susceptibility with high confidence, and thorough pre- and post-test counselling for the individual. It is well recognised however that in focusing on high-risk families, often identified through an affected individual presenting to clinical genetics clinics, around 50% of mutation carriers are missed [7, 8]. In response, testing for *BRCA1/2* mutations in breast cancer and mismatch repair (MMR) deficiency in colorectal cancer has been “mainstreamed” into oncology services, with the hope of improving diagnosis and care of individuals and their families [17]. Cancer screening, conversely, centres around finding early disease and removing it effectively in the general population. Typically, the screening test itself (for example mammography or faecal stool testing) has a low specificity (and consequently low positive predictive value, PPV) and high sensitivity (high negative predictive value, NPV). This allows it to capture as many cases as possible but at the cost of a high false-positive rate. This is then traditionally followed by a diagnostic test which has a high sensitivity, specificity, PPV and NPV (for example breast biopsy or colonoscopy) [16]. Thus far, universal tumour screening (UTS) for MMR status in CRC tumours is the first instance of population-based screening in the UK for a CSG (the defined population being CRC patients) [18]. The process is aligned to the traditional screening paradigm of lower specificity screening test (MMR testing), followed by a separate process of informed consent for the genetic test in which MMR genes are sequenced. Beyond UTS, population-based screening for cancer risk susceptibility in the general population may in fact utilise a diagnostic test for CSGs (with high PPV and NPV), rather than a screening test, posing a further challenge to traditional paradigms [16]. In addition, the purpose of adding CSGs or PRS to cancer screening programmes shifts the aim of the programme from early diagnosis to risk prognostication—with far wider implications for personal and familial health.

With the increasing drive for “personalised” healthcare encompassing prevention as well as medical management, there is a need to synthesise concepts of these two differing approaches-individual genetic testing and population health approaches of screening-incorporating CSGs and PRS. Cancer genetics is at the forefront of researching and evaluating population screening and is likely to be held as an exemplar for implementation, laying the path for other disease areas where screening may be beneficial. Here, we will consider the frameworks used to evaluate organised screening programmes and examine how population-based CSG and PRS-based screening hold up to the evaluation against current frameworks. We will go on to discuss the complexity of using a diagnostic test as a screening test and consider whether current frameworks and guides are appropriate for or can accommodate this developing area of population health.

## How Should we Evaluate Population-Based Genetic Screening?

Organised population-based screening programmes are expected to meet a set of standards prior to implementation. In particular, national screening programmes are held to rigorous account. In 1968, Wilson and Jungner published a seminal paper detailing 10 criteria that should be met for implementation of screening for disease [19]. These principles have been widely used and, over time, many revised criteria have been proposed [20, 21••]. Despite this guidance, screening decisions continue to be challenging and at times, controversial [22], and the complexity of evaluating genetic testing in this context has been recognised for some time.

In an attempt to synthesise the many guidelines now in existence, Dobrow and colleagues derived consensus guidelines for screening through a systematic review and Delphi consensus process [21••]. They identified 12 general principles falling into three categories: disease/condition, test/intervention and programme/system. Whilst eight of these principles overlap with the Wilson and Jungner criteria, four new principles emerged, three of which (screening programme acceptability and ethics, screening programme benefits and harms, screening programme quality and performance management) fall into the programme/system category, around which there is increasing focus in more recent guidance. This shift in priorities reflects broader changes including a shift towards informed choice, accountability of policymakers and cost-effectiveness in the context of universal healthcare systems [20]. In the UK, screening programmes are evaluated according to the “Criteria for appraising the viability, effectiveness and appropriateness of a screening programme” from Public Health England (PHE) [23], which incorporates many of these programme-based considerations (see Table 1 for comparison). Of note, this makes specific reference to genetics, stating:

**Table 1:** Comparison of evaluative frameworks: Dobrow’s consolidated screening principles [21••], Public Health England Screening evaluation criteria [23] and ACCE framework for review of genetic testing [24]

Domain	Dobrow screening principles		Public Health England Criteria	ACCE framework questions
Condition	Epidemiology	“The epidemiology of the disease or condition should be adequately understood, and the disease or condition should be an important health problem”	“Condition should be an important health problem […]. Epidemiology, incidence, prevalence […] of the condition should be understood…”	“What is the specific clinical disorder to be studied?” “What is the prevalence of the disorder in this setting?”
	Natural history	“The natural history of the disease or condition should be adequately understood […] and there should be a clear pre-clinical phase”	“Natural history of the condition should be understood” “If the carriers of a mutation are identified as a result of screening the natural history of people with this status should be understood, including the psychological implications”	“What are the clinical findings defining this disorder?” “What is the natural history of the disorder?”
	Target population	“The target population for screening should be clearly defined (e.g. with an appropriate target age range), identifiable and able to be reached”	Target population not explicitly considered, though referenced in test domain	Target population not explicitly considered
		Other primary prevention measures not explicitly considered	“Cost-effective primary prevention interventions should have been implemented as far as practicable”	Other primary prevention measures explicitly not considered
Test	Test performance	“Screening test performance should be appropriate for the purpose, with all key components specific to the test (rather than the screening program) being accurate (e.g. in terms of sensitivity, specificity and positive predictive value) and reliable or reproducible. The test should be acceptable to the target population and it should be possible to perform or administer it safely, affordably and efficiently.”	“There should be a simple, safe, precise and validated screening test” “The test, from sample collection to delivery of results, should be acceptable to the target population”	“What DNA test(s) are associated with this disorder?” “Are preliminary screening questions employed?” “Is it a stand-alone test or is it one of a series of tests? If it is part of a series… are all tests performed in all instances (parallel) or are only some tests performed on the basis of other results (series)?” “Is the test qualitative or quantitative?” “How often is the test positive when a mutation is present (sensitivity)?” “How often is the test negative when a mutation is not present (specificity)?” “Is an internal QC program defined and externally monitored?” “Have repeated measurements been made on specimens?” “What is the within- and between-laboratory precision?” “If appropriate, how is confirmatory testing performed to resolve false positive results in a timely manner?” “What range of patient specimens have been tested?” “How often does the test fail to give a useable result?” “How similar are results obtained in multiple laboratories using the same, or different technology”
	Test interpretation	“Screening test results should be clearly interpretable and determinate (e.g. with known distribution of test values and well-defined and agreed cut-off points) to allow identification of the screening participants who should (and should not) be offered diagnostic testing and other postscreening care”	“The distribution of test values in the target population should be known and a suitable cut-off level defined and agreed”	“How often is the test positive when the disorder is present (sensitivity)?” “How often is the test negative when a disorder is not present (specificity)?” “Are there methods to resolve clinical false positive results in a timely manner?” “Has the test been adequately validated on all populations to which it may be offered?” “What are the positive and negative predictive values?” “What are the genotype/phenotype relationships?” “What are the genetic, environmental or other modifiers?”
		Genetic testing/reviewing of variants not explicitly considered	“If the test is for a particular mutation or set of genetic variants the method for their selection and the means through which these will be kept under review in the programme should be clearly set out”	Review of genetic variants not explicitly considered
Intervention	Postscreening options	“There should be an agreed on course of action for screening participants with positive screening test results that involves diagnostic testing, treatment or intervention, and follow-up care that will modify the natural history and clinical pathway for the disease or condition that is available, accessible and acceptable to those affected; and that results in improved outcomes …”	“There should be an agreed policy on the further diagnostic investigation of individuals with a positive test result and on the choices available to those individuals” “There should be an effective intervention, with evidence that intervention at presymptomatic stage leads to better outcomes” “There should be agreed evidence based policies covering which individuals should be offered interventions” “Clinical management of the condition and patient outcomes should be optimised in all health care providers prior to participation in a screening programme”	“What is the impact of a positive (or negative) test on patient care?” “If applicable, are diagnostic tests available?” “Is there an effective remedy, acceptable action, or other measurable benefit? Is there general access to that remedy or action?” “What are the results of pilot trials?” “What health risks can be identified for follow-up testing and/or intervention?”
Programme	Infrastructure	“There should be adequate existing infrastructure […] or a clear plan to develop adequate infrastructure, that is appropriate to the setting to allow for timely access to all components of the screening program”	“Adequate staffing and facilities […] should be available prior to the commencement of the screening programme”	“What is the clinical setting in which the test is to be performed?” “What facilities/personnel are available or easily put in place?”
	Coordination	“All components of the screening program should be coordinated and, where possible, integrated with the broader health care system…”	“There should be a plan for managing and monitoring the screening programme”	“What methods exist for long term monitoring?”
	Acceptability and ethics	“All components of the screening program should be clinically, socially and ethically acceptable to screening participants, health professionals and society, and there should be effective methods for providing screening participants with informed choice, promoting their autonomy and protecting their rights.”	“Evidence the complete screening programme is clinically, socially and ethically acceptable to health professionals and the public” “Evidence-based information […] should be made available to potential participants to assist them in making an informed choice” “Public pressure for widening the eligibility criteria, for reducing the screening interval, and for increasing the sensitivity of the testing process, should be anticipated. Decisions […] should be scientifically justifiable to the public”	“Is the test being offered to a socially vulnerable population?” “What educational materials have been developed and validated and which of these are available?” “Are there informed consent requirements?” “What is known about stigmatisation, discrimination, privacy/confidentiality and personal/family social issues?” “Are there legal issues regarding consent, ownership of data and/or samples, patents, licencing, proprietary testing, obligation to disclose, or reporting requirements?” “What safeguards have been described and are these safeguards in place and effective?”
	Benefits and harms	The expected range and magnitude of benefits […] and harms […] for screening participants and society should be clearly defined and acceptable, and supported by existing high-quality scientific evidence (or addressed by ongoing studies) that indicates that the overall benefit of the screening program”	“There should be evidence from high quality randomised controlled trials that programme reduces morbidity and mortality” “The benefit gained by individuals should outweigh harms”	“Is there an effective remedy, acceptable action, or other measurable benefit? Is there general access to that remedy or action?”
	Economic evaluation	“An economic evaluation […] of the screening program, using a health system or societal perspective, should be conducted (or a clear plan to conduct an economic evaluation) to assess the full costs and effects of implementing, operating and sustaining the screening program while clearly considering the opportunity costs and effect of allocating resources to other potential nonscreening alternatives”.	“The opportunity cost of the screening programme […] should be economically balanced in relation to healthcare expenditure as a whole” “All other options for managing the condition should have been considered (such as improving treatment or providing other services), to ensure that no more cost effective intervention”	“What are the financial costs associated with testing?” “What are the economic benefits associated with actions resulting from testing?”
	Quality and performance	“The screening programme should have clear goals or objectives that are explicitly linked to program planning, monitoring, evaluating and reporting activities, with dedicated information systems and funding, to ensure ongoing quality control and achievement of performance targets”	“There should be a plan for managing and monitoring the screening programme and an agreed set of quality assurance standards”	“What quality assurance measures are in place?” “What guidelines have been developed for evaluating program performance?”

“If the carriers of a mutation are identified as a result of screening, the natural history of people with this status should be understood, including the psychological implications”

and

“If the test is for a particular mutation or set of genetic variants the method for their selection and the means through which these will be kept under review in the programme should be clearly set out”.

## Evaluation of Population CSG and PRS Screening by Existing Screening Criteria

Taking as a base for evaluation Dobrow’s 12 consolidated screening principles (highlighted in bold in the text below), does population-based screening for CSGs, and polygenic risk stratification, fulfil these screening principles? This is heavily predicated on the form and structure of a cancer genetic population screening programme. Population screening for CSGs will have a different profile to risk stratification of existing screening programmes, unless of course the two operate in unison (the case for combined assessment being supported by the modification of monogenic risk by polygenic risk [5••, 25]). In addition, a model in which samples are taken in adulthood and disposed of after testing of a limited number of CSGs would likely be less challenging, though potentially less effective, than a model in which a sample is taken in early childhood, tested for a wide range of CSGs and common risk loci and stored across the life course. Here, we will consider the general principles, presuming screening in adulthood.

### Disease/Condition Principles Domain

Screening for CSGs would rely on our **understanding of the epidemiology and natural history** of these conditions which is currently derived from studies of high-risk families identified through cancer genetics clinics. It is highly likely that the population penetrance and phenotype differ from this. For example, unbiased population estimates of penetrance for medullary thyroid cancer of p.Val804Met mutations in the *RET* oncogene were far lower than estimated in carrier-based analysis [26]. Conversely, the penetrance of *PALB2* mutations for breast cancer is far greater than initially estimated [1••, 27]. Understanding these emerging disease patterns is essential to accurately inform ongoing risk information, stratification and preventative recommendations. Recognising that we will only obtain this knowledge through detection and analysis of population cases identified through screening initiatives, with associated comprehensive research programmes [1••], we assert that this should not be a barrier to implementation.

The **target population**, and in particular, the age range for CSG screening, is also an area of considerable debate. A screening programme that includes high-penetrance CSGs could arguably begin at 20–30 years of age to maximise opportunity for early screening, prevention and behavioural risk reduction, however, it could also be argued that adding genetic testing onto existing screening programmes is likely to facilitate uptake. Further still, some have argued for incorporation of testing into the Newborn Screening Programme (NBS), which might improve uptake [28], however genetic testing of children has historically been performed for diseases with high childhood risk of significant morbidity or mortality; the ethical and legal implications of this approach will be discussed below. One option might be to test alongside the NBS, but release results only when they become clinically relevant, and with an individual’s consent [16].

### Test/Intervention Domain

Genetic testing, including targeted sequencing, genome sequencing, and genotyping arrays, are all utilised clinically at present, and their **performance characteristics** (i.e. analytical validity) are established. However, acceptability to the target population and the ability to perform testing affordably and efficiently still needs to be evaluated at a population level.

Though use of NGS in healthcare is widespread, careful consideration would need to be given to the **interpretation of test results**. Establishing pathogenicity of many variants in CSGs has been difficult, particularly for low-frequency variants and those associated with moderate risk, though techniques are improving [1••]. This results in a large number of variants of unknown significance, comprehensive guidelines for management and communication of which are currently lacking [29]. As evidence evolves and variants are reclassified, there is a potential need to recontact patients with relevant information; this already occurs in cancer genetics in the UK, but would be a new step for a screening programme [30]. Recent European guidelines endorse the practice, where there is clinical and personal utility, and note requirements for sustainable recontacting procedures and shared responsibility [31]. The organisation and ethics of recontacting is an evolving field, and doing so at population scale will add additional complexity. Similarly, as risk models become more sophisticated, these might also need to be updated and individuals re-categorised. (It is likely, however, that updated risk estimates derived from new PRS would not dramatically affect risk categorisation, given the likely small effect size [5••]). Of note, Dobrow et al. state that “screening test results should be clearly interpretable […] to allow identification of screening participants who should (and should not) be offered diagnostic testing” [21••]. As noted above, in the case of CSGs, the “screening” test is the diagnostic test.

Guidance for **post-screening test options**, including risk estimates and recommendations for management, exist for high-penetrance CSGs through existing guidelines, but are based on risk estimates from a select population. Evidence for moderate penetrance genes is more variable and based on expert opinion [32], in part as relative risks and phenotypes for these genes are less well understood [1••]. It is therefore likely that some individuals would initially be overtreated at initiation of population screening. As our understanding of population penetrance evolves, alongside understanding of genotype/phenotype correlations, pathogenicity of newly identified genes and the effects of risk modifiers, risk estimates and management strategies will also evolve. Prompt review of evidence and translation into guidance would be essential if implementing screening at a population level. In stratified screening, the optimal approach (e.g. higher intensity screening for high risk vs. lower intensity or no screening for low risk), and the optimal risk thresholds, will depend on a range of factors including relative risks, rates of overdiagnosis, and costs, and are, and will need to remain, areas of ongoing research [11, 14].

### Programme/System Principles

The **screening programme infrastructure** required to run a population screening programme is considerable, including test facilities, human resources, information technology, and in the case of genomics, vast data storage capacity [21••]. Whilst scaling-up of genomic testing has begun through the development of genomic medicine centres, significant further investment in infrastructure and training would be needed to facilitate population-level screening for high-penetrance CSGs, and/or risk-based stratification of existing screening cohorts [14]. Both genetic technologies and our understanding of the genetic basis of disease are changing rapidly. There will be a need to build real-time learning into screening programmes to be able to quickly re-appraise the information, allowing programmes to keep pace in a cost-effective and timely manner.

Screening should ideally be **integrated** into the healthcare system more broadly. Within the UK, **screening programme coordination and quality and performance management** is overseen by the National Screening Committee of Public Health England. Screening programme coordination typically involves “recruitment, testing, information access, referral, follow-up, patient education and support, staff training, and management and evaluation” [21••]. Screening for genetic risk has additional complexities, with issues including re-evaluation of results, subsequent cascade testing, ensuring appropriate follow-up as well as staff training and capacity. Both the infrastructure and processes for these issues will need careful consideration.

Dobrow states that a programme should be “**clinically, socially and ethically acceptable** to screening participants, health professionals and society, and there should be effective methods for providing screening participants with informed choice, promoting their autonomy and protecting their rights”.

The evidence for acceptability of screening for CSGs is positive to date. Several recent trials have suggested public support for population genetic testing for CSGs or screening stratified by PRS [9, 33–35]. However, a consequent reduction in screening for low-risk groups was a concern to those questioned [34]. In one questionnaire-based study of screening stratified by PRS just 27%, of almost 3000 individuals, said they would accept less frequent screening if considered low risk [35]. Thus it seems unlikely that this approach would gain public approval [5••], and this may prove a barrier to maximising the efficacy and cost-effectiveness of such programmes. It is notable that acceptability is likely to vary depending on both the framing of the purpose for population screening—for example as benefit to patients rather than cost-effectiveness [36]--and on the context of both the intervention and the population in question [37].

**Screening programme harms and benefits** for both individuals and society, supported by high-quality evidence, should demonstrate overall benefit [21••]. We will explore the ethical issues using the four traditional principles of medical ethics (autonomy, beneficence, non-maleficence and justice [38]) whilst drawing attention to the limitations of using this framework in a population context.

In the screening context, the principle of autonomy arises in the timing of testing and the process of informed consent. In current clinical practice, whilst it is considered preferable to test for highly penetrant adult-onset disorders in adulthood, justified by the need to respect autonomy, childhood testing does occur. Expert consensus regarding childhood testing is that adopting a position of caution, supporting families and considering guidelines as a framework for decision-making is the optimal approach [39]. This is a nuanced area and the acceptability of childhood testing is highly context-dependent [40]. Given these reservations, it seems unlikely that population-based screening programmes incorporating genomic information will be implemented in childhood in the foreseeable future.

The process of informed consent is often advocated as another way to try to ensure individual autonomy. Population testing of CSGs and incorporating PRS should therefore include information on risk assessment (and uncertainties around risk), health implications of a positive result, data issues (including storage, access and use in research), potential for incidental findings, relevance for family members, possibility of re-contact and implications for work and insurance if relevant [14, 36, 41].

In the context of using population-based genotyping to stratify existing screening programmes, Hall and colleagues have made an argument for an alternative normative framework, recognising that personal autonomy ought not to be central to decision-making [28]. Whilst Hall et al. make their claim with respect to including genotyping in newborn screening programmes, the argument remains valid for using population-based genotyping to stratify existing screening programmes. Polygenic risk is far less predictive of individual risk, with lower impact on family members. Benefits of such an approach would include increased coverage through integration with existing programmes, increased opportunities for primary prevention and avoidance of relevant risk factors, noting the need for data to be handled in such a way as to maximise confidentiality and privacy [28].

Overall benefits of screening (beneficence) for both high-risk CSGs and of population stratification are considerable for both the individual and the population. Identification of CSGs facilitates understanding of disease risk for an individual and their family, affords the opportunity for risk reduction through behaviour change (though evidence for this is currently poor [42, 43]) and appropriate screening and prophylaxis with subsequent reductions in morbidity and mortality [44]. These may all be extended to family members through cascade testing [8]; of note, PHE screening guidelines make reference to this, stating “evidence relating to wider benefits of screening, for example those relating to family members, should be taken into account where available” [23]. In addition, the personal utility and empowerment of health ownership through genetic knowledge may be considerable, though there is a tension with the difficulties and provider responsibility of accurately explaining potentially unvalidated or uncertain risks [15]. Risk stratification may similarly have considerable personal utility [34, 35], and may improve benefit-to-harm ratio by reducing overdiagnosis in low-risk individuals [11].

Screening programmes need to consider evidence for psychological harm (malevolence) as a result of their screening testing. Evidence within the context of genetic testing for CSGs is limited, but suggests relatively low levels of psychological harm [45]; higher quality studies are needed to provide sufficient reassurance. As described above, uncertainties around true penetrance for monogenic CSGs in the unaffected population could result in over-screening and unnecessary preventive surgery [46]. Stigmatisation and discrimination against those diagnosed with a high-risk CSG is also a concern, for which legal protections have been put in place [47]. Concerted ongoing efforts to improve genetic literacy in the general population, industries and public services is required to insure against this harm [28].

Distributive justice suggests that varied societal groups should have equal access and chance of benefit from intervention [36]. Population screening for CSGs could improve access by bypassing diagnoses made through clinical genetics, which are less well accessed by minority groups [48, 49]. However, it is well recognised that screening uptake is also poorer in minority and lower socioeconomic groups [50, 51], and approaches for tackling this should be considered in the set-up of any screening programme. Furthermore, in polygenic risk, where estimates are derived from risk estimates from European populations, transferability to non-European populations is limited [52]; use of PRS risk stratification therefore has the potential to widen health inequalities in the absence of non-European risk estimates [5••, 36]. This applies too to the high-penetrance setting, where penetrance and phenotypes may vary in different populations [53, 54]. It should also be noted that personal utility, ethical considerations and risk/benefit considerations will also be affected by cultural and social background. These issues must be acknowledged and steps taken to remedy the resulting disparities.

**Economic evaluation** is essential in a public health programme, and in the current restrictive financial climate, the extensive investment required for population genetic screening would need to be well justified. For breast cancer, BRCA screening in the context of high-risk populations has already been shown to be cost-effective in the USA and UK [10, 55], and recent evidence has shown screening for hereditary breast and ovarian cancer genes in an unselected population to be cost-effective over testing based on clinical or family history criteria [44]. In addition, a recent cost-effectiveness evaluation of early adulthood screening for *BRCA1/BRCA2/MLH1/MSH2* with carrier screening for cystic fibrosis, spinal muscular atrophy and fragile X syndrome in the Australian single-payer system found this approach to be highly effective in preventing cancer deaths and highly cost-effective [56]. Polygenic risk-informed cancer screening has also been shown to be cost-effective in breast cancer [11, 57], with evaluations in colorectal and prostate cancer underway. Initial evidence of the cost-effectiveness of utilising genomic testing within population screening ought to drive interest in maximising this potential within publicly funded healthcare systems.

## Discussion

We have outlined the principles used to evaluate screening programmes and examined how population-based CSG and PRS-based screening holds up to evaluation against these. A particular complexity to the introduction of genomic testing to screening programmes, particularly CSGs, is that a diagnostic test is used as a screening test. Might the frameworks used for evaluating diagnostic genetic tests offer a fitting alternative for evaluating the use of genomic information in screening programmes?

In contrast to the screening guidelines outlined above, in a systematic review of evaluative frameworks for genetic tests, Pitini and colleagues [58] identified 29 different frameworks for the evaluation of genetics tests in their recent systematic review, of which the ACCE framework was the most commonly used (*n* = 13). In the UK, the ACCE framework was used to guide implementation of genetic testing through the UK Genetic Testing Network [59–61]. Health technology assessments were the next most common (*n* = 6). Pitini and colleagues also found a marked paucity of context-related evaluation, namely delivery models, economic assessment, and organisational implications (though these are well described in HTA-based models). Evaluation studies are essential components of public health delivery (including screening), particularly in the context of a publicly funded care system such as the National Health Service, where resources are ever-constrained and efficiency is key.

The ACCE framework most commonly found in genetic testing evaluations was initially proposed by the US Centres for Disease Control and Prevention [24] and is a set of 44 questions evaluating analytic validity, clinical validity, clinical utility and ethical, legal and social implications. **Analytic validity** describes the sensitivity and specificity of the test involved, with additional metrics such as assay robustness and quality assurance often also considered. In the context of cancer genetics, **clinical validity** considers four main questions [62]: Are genetic variants in the gene associated with cancer risk? Which variants are associated with risk? How big is the risk conferred? How have the risks been estimated?

**Clinical utility** typically comprises the associated risks and benefits and utility in terms of health outcomes. Some frameworks expand the concept of patient benefit to include personal utility, that is, individual benefit to patients such as better understanding or behavioural choices [58]. This expansion may be particularly relevant in genetics, where genetic information changes peoples’ lives, and that of their families, rather than a specific disease course [15]. In the context of a population screening programme, it is not clear that personal utility appropriately encompasses all of the utility of the intervention delivered to the population.

**Ethical, legal and social implications** are a prominent feature of public awareness of genetic testing and gain even greater importance both for the public and for policymakers when considering population-based testing [41]. Outcomes considered must be meaningful to patients, their families, and communities, and so expand far beyond those traditionally considered in screening evaluations [15, 63, 64]. In addition, the outcomes of relevance are guided by the scenario under consideration (i.e. are context-driven) [63]. In the context of population testing, some of these measures, such as cascade testing and health utilisation for family members, or risk of discrimination, may be more difficult to evaluate, but certainly require careful consideration.

Both Wilson and Jungner-derived screening criteria, and ACCE-based assessment of genetic testing, have some relevance in the evaluation of CSG- and PRS-based screening. Mapping of this framework to those from Dobrow et al. and PHE [21••, 23] is shown in Table 1, highlighting differences in each approach. It is likely that evaluation of population screening incorporating genomics requires the integration of these approaches or introduction of new evaluation methods.

## Conclusion

The development of effective and acceptable population screening programmes for CSGs or the inclusion of polygenic risk in existing screening programmes clearly poses a wide array of challenges. Despite the hype around genomic and personalised medicine approaches [65, 66] and the “rhetoric of empowerment” in much of the public dialogue [42], implementation of these techniques on a population level needs to be fully informed and rigorously evaluated.

Screening decisions are ultimately political but should be informed by best available evidence and the values of the population concerned [20, 67]. The evidence base in genetics is rapidly evolving, and evaluation of population approaches to genetic testing will clearly be highly complex, given the breadth of outcomes for genetic testing, difficulty in measuring these, and variation in individual values. These broader considerations shift the risk/benefit considerations of traditional screening away from morbidity and mortality (though these are still important) towards personal and population utility for both patients, their families and the wider population which may be of equal value. As we have outlined here, the existing frameworks for evaluation of screening programmes will need to be modified to incorporate the additional complexities of genetic testing. Implementation would require significant financial, infrastructural, educational, and political investment, and ongoing multidisciplinary research alongside clinical implementation will be essential to ensure well-informed ongoing care and to improve the knowledge based upon which this care is built. In the context of a cancer screening programme, prior evidence of clinical validity is required by all frameworks, however understanding of true prevalence and penetrance of CSGs may only be obtainable through population-based evaluation, perhaps necessitating post-implementation (re-)evaluation.

These complexities and hurdles are not however insurmountable. Clinically- and cost-effective management of the high cancer risk population depends on identifying these individuals, and the ability to provide accurate evidence-based management advice. Given the significant numbers with CSG mutations undetected in the population, our rapidly expanding evidence for managing them and evidence of cost-effectiveness, now is the time to develop an appropriate evaluative framework for population-based approaches incorporating genomic testing, and to begin the public dialogue required to inform decision making and policy implementation.
